# Administration of Non-Absorbable Antibiotics to Pregnant Mice to Perturb the Maternal Gut Microbiota Is Associated with Alterations in Offspring Behavior

**DOI:** 10.1371/journal.pone.0138293

**Published:** 2016-01-20

**Authors:** Shiro Tochitani, Takahiro Ikeno, Tatsuhito Ito, Asuka Sakurai, Tomoki Yamauchi, Hideo Matsuzaki

**Affiliations:** 1 Division of Development of Mental Functions, Research Center for Child Mental Development, University of Fukui, Fukui 910–1193, Japan; 2 Division of Developmental Higher Brain Functions, Department of Child Development, United Graduate School of Child Development, Osaka University, Kanazawa University, Hamamatsu University School of Medicine, Chiba University and University of Fukui, Osaka 565–0871, Japan; 3 Research and Education Program for Life Science, University of Fukui, Fukui 910–8507, Japan; 4 School of Medicine, Faculty of Medical Sciences, University of Fukui, Fukui 910–1193, Japan; University of Arkansas for Medical Sciences, UNITED STATES

## Abstract

There is increasing evidence that the gut microbiota plays a major role in host health and disease. In this study, we examined whether perturbation of the maternal gut microbiota during pregnancy, induced by administration of non-absorbable antibiotics to pregnant dams, influences the behavior of offspring. Terminal restriction fragment length polymorphism analyses of fecal bacterial composition showed that the relative abundance of the bacterial order *Lactobacillales* was lower in offspring born from antibiotic-treated dams (20.7±3.4%) than in control offspring (42.1±6.2%) at P24, while the relative abundance of the bacterial family *Clostridium* subcluster XIVa was higher in offspring born from antibiotic-treated dams (34.2±5.0%) than in control offspring (16.4±3.3%). Offspring born from antibiotic-treated dams exhibited low locomotor activity in both familiar and novel environments, and preferred to explore in the peripheral area of an unfamiliar field at postnatal week 4. At postnatal weeks 7–8, no difference was observed in the level of locomotor activity between control offspring and offspring from antibiotic-treated dams, while the tendency for the offspring from antibiotic-treated dams to be less engaged in exploring the inside area was still observed. The behavioral phenotypes of the offspring from antibiotic-treated dams at postnatal week 4 could be rescued to a considerable extent through fostering of these offspring by normal dams from postnatal day 1. Although the detailed underlying mechanisms are not fully elucidated, the present results suggest that administration of non-absorbable antibiotics to pregnant dams to perturb the maternal gut microbiota during pregnancy leads to alterations in the behavior of their offspring.

## Introduction

Many lines of evidences indicate that the gut microbiota plays a major role in host health and disease [1, 2]. The gut microbiota is involved in immunomodulation, drug disposition and detoxification, nutrition, and metabolism [1, 2], and also interacts with the central nervous system, possibly through neural, endocrine, and immune pathways, to influence host brain functions and behaviors [3–8]. There is also emerging evidence that the gut microbiota contributes to host brain development. Germ-free mice display increased hyperactivity and reduced anxiety compared with specific pathogen-free (SPF) mice with normal gut biota [9]. Germ-free mice also exhibit robust social impairments including social avoidance and diminished preference for social novelty [10]. Depletion of the gut microbiota by applying antibiotics (AB) to adolescent mice from weaning was shown to reduce anxiety and induce cognitive deficits [11]. Germ-free pregnant mice have fetuses that show increased permeability of the blood-brain barrier, and this increased permeability is also observed in adult germ-free mice [12]. To explore whether and how the function of the maternal gut microbiota during pregnancy contribute to the brain development of offspring, we applied non-absorbable AB to pregnant mice to perturb the gut microbiota for the period of embryonic day (E) 9 to E16 and performed behavioral analyses on their offspring. Our findings showed that administration of non-absorbable AB to pregnant dams to perturb the gut microbiota during pregnancy leads to alterations in the behavior of their offspring.

## Materials and Methods

### Ethics statement

The methods described in this manuscript were carried out in accordance with approved ethics guidelines. All animal manipulations were performed in accordance with the National Institute of Health Guide for the Care and Use of Laboratory animals and were approved by the Animal Research Committee of University of Fukui.

### Animals and non-absorbable AB treatment

**Fig 1:**
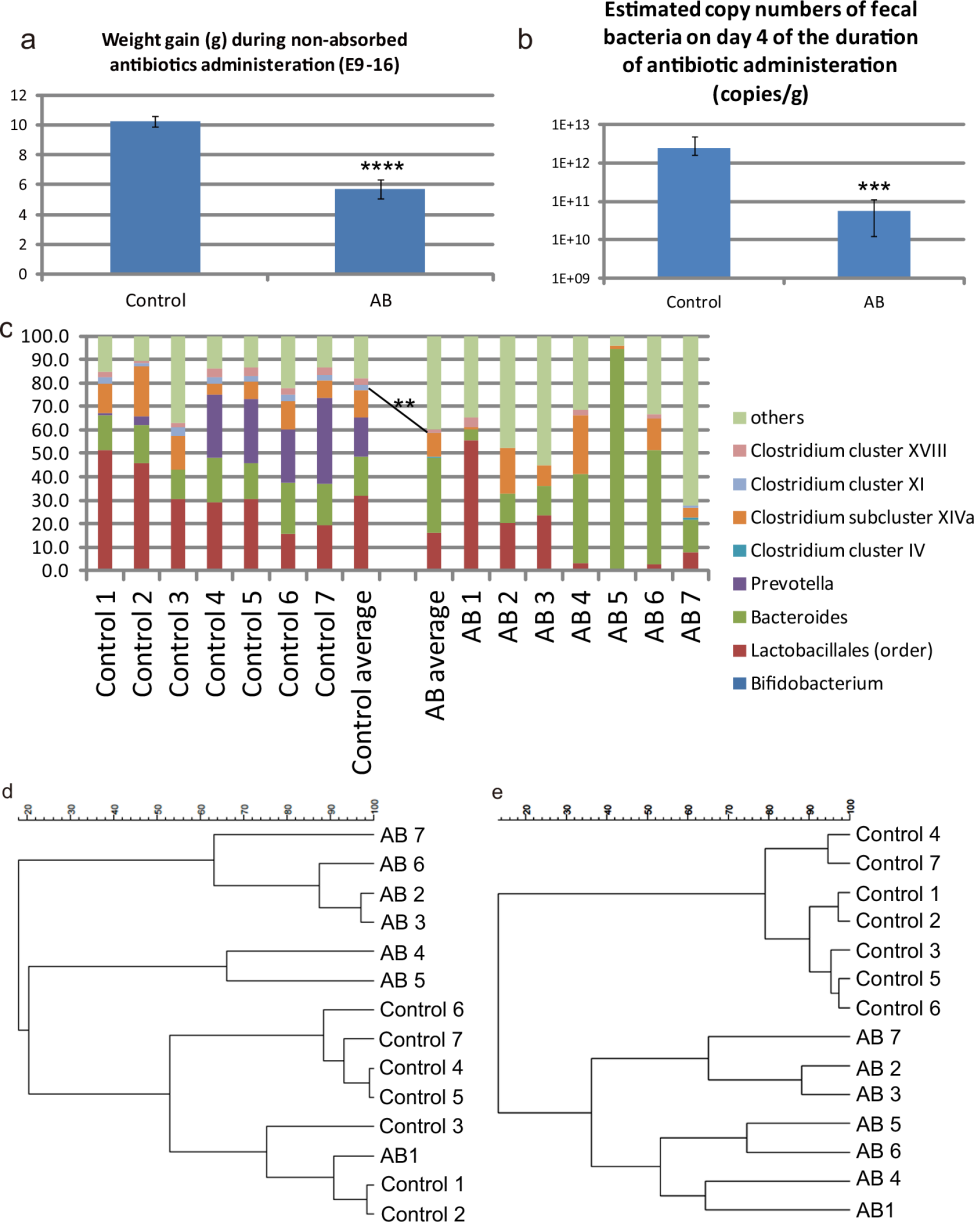
Effects of administration of non-absorbable AB to pregnant dams. (**a**) Weight gain (g) of dams during the period of non-absorbable AB administration (E9–E16). Control, n = 20; AB-treated mice, n = 18. ****P<0.0001 (*t*-test). (**b**) Copy numbers of 16S ribosomal RNA genes in fecal samples obtained on day 4 of the 7-day AB administration period (copies/g); n = 7 for both groups. ***P = 0.0006 (Mann-Whitney test). (**c**) Bar charts showing the taxonomic profiles of fecal bacteria, as determined by T-RFLP analysis, for fecal samples from seven control mice (Control 1–7) and seven AB-treated mice (AB 1–7). Fecal samples analyzed of each group were the same samples as those analyzed in (**b**). Relative abundance of Clostridium cluster XI was lower in AB-treated mice than in control mice. **P = 0.0014 (Mann-Whitney test). (**d**) Cluster analysis of the fecal microbiota composition, as determined by T-RFLP analysis, performed by Pearson correlation and UPGMA. The control mice and AB-treated mice are principally grouped in different clusters. (**e**) Cluster analysis of the fecal microbiota composition, as determined by T-RFLP analysis, obtained with Dice coefficient and the Ward algorithm.

**Fig 2:**
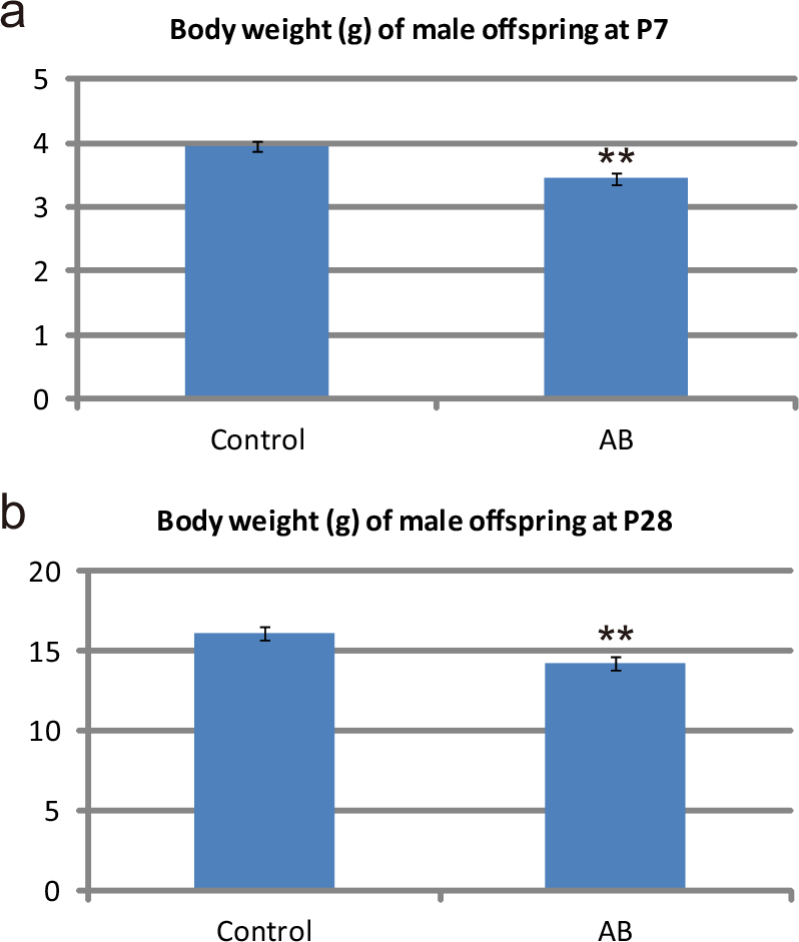
Body weight of offspring after administration of non-absorbable AB to pregnant dams. (**a**) Body weight at P7 in male offspring from control (control offspring; Control) and offspring from AB-treated dams (AB offspring; AB). **P = 0.0023 (Mann–Whitney test); control, n = 27 from 7 dams; AB, n = 15 from 4 dams. (**b**) Body weight at P28 in male control offspring (Control) and AB offspring (AB). **P = 0.0057 (*t*-test); control, n = 15 from 4 dams; AB, n = 9 from 3 dams.

**Fig 3:**
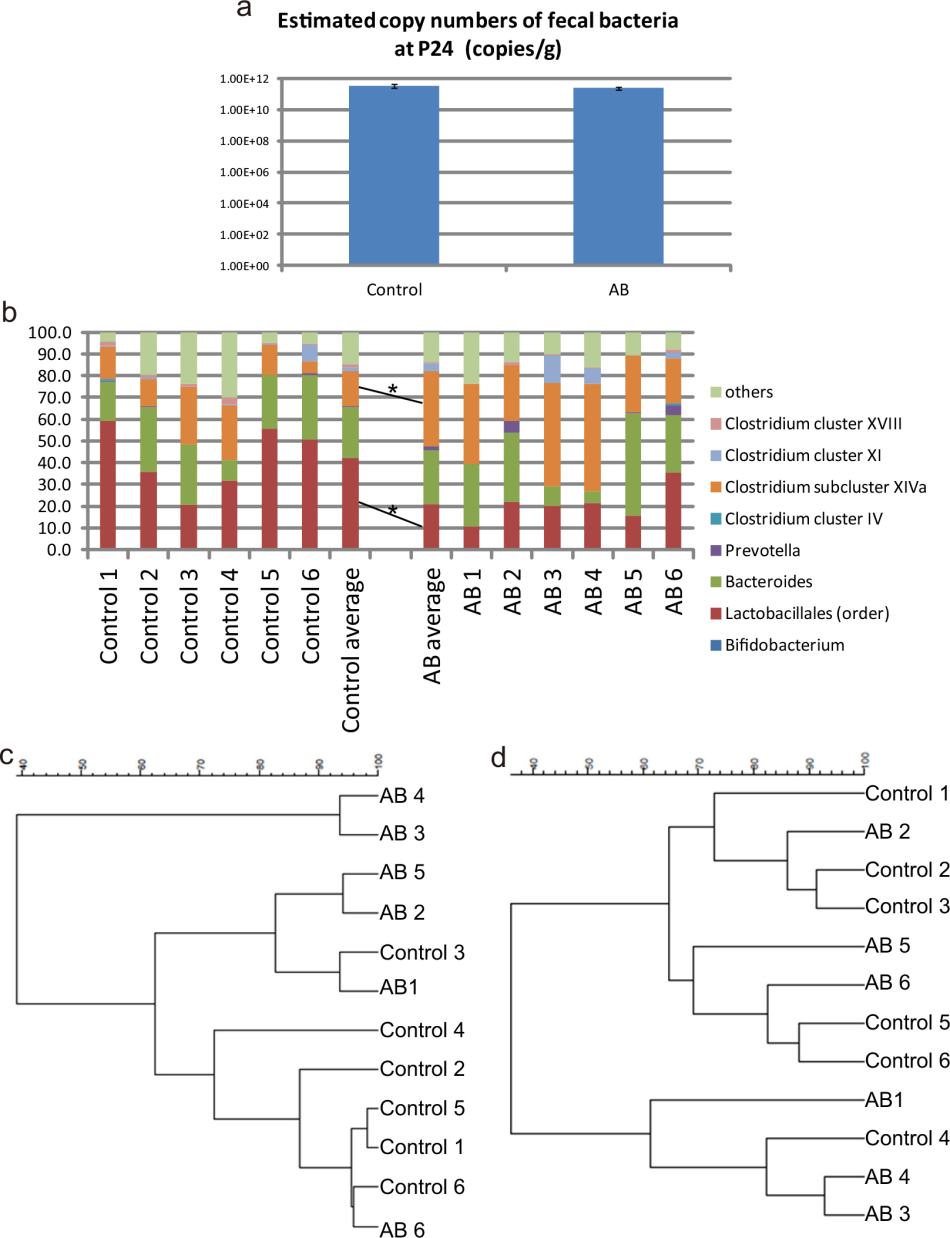
Gut microbiota of offspring after administration of non-absorbable AB to pregnant dams. (**a**) Mean 16S ribosomal RNA gene copy numbers in fecal samples (copies/g); n = 6 per group. Fecal samples were collected from cages housing 2–4 male offspring. (**b**) Bar charts showing the taxonomic profiles of fecal bacteria, determined by T-RFLP analysis, which were obtained for fecal samples from six groups of control offspring (Control 1–6) and six groups of AB offspring (AB 1–6). Fecal samples analyzed of each group were the same samples as those analyzed in (**a**). The relative abundance of *Lactobacillales* is lower in AB offspring than in control mice, while that of Clostridium subcluster XIVa is higher in AB offspring than in control mice. *^1^P = 0.0127, *^2^P = 0.0132 (*t*-test). (**c**) Cluster analysis of the fecal microbiota composition, as determined by T-RFLP analysis, performed by Pearson correlation analysis and UPGMA. The control offspring and AB offspring are principally grouped into different clusters. (**d**) Cluster analysis of the fecal microbiota composition, as determined by T-RFLP analysis, obtained by Dice coefficient analysis and the Ward algorithm. The groups of control offspring and AB offspring do not appear to be divided into different clusters.

**Fig 4:**
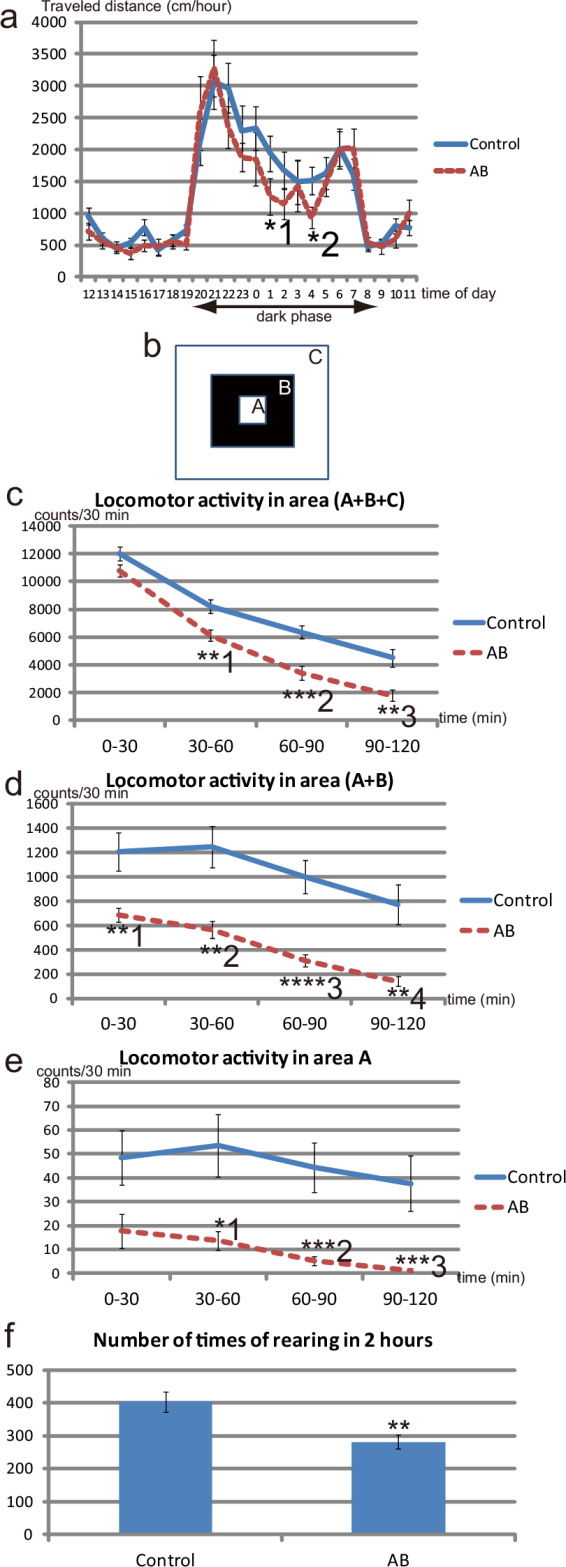
Behaviors of AB offspring at PW4. (**a**) Data for 24-h cycle activity in a familiar environment for control offspring and AB offspring at P28–P32. Throughout the dark phase (20:00 to 8:00), the mean activity of AB offspring is generally lower than that of control offspring. Significant differences are detected for 01:00–01:59 (*^1^P = 0.043, Mann–Whitney test) and 04:00–04:59 (*^2^P = 0.0362, Welch’s corrected *t*-test). Control, n = 29 from 8 dams; AB, n = 18 from 5 dams. (**b**) Schematic diagram showing the areas in the open field. In the entire field, which is a square of 48 cm per side, two nested squares (7.8-cm square and 24.6-cm square) are assumed. Area A (white square in the center of the field), area B (black area), and area C are then defined so that the three areas do not overlap with one another. (**c**) Locomotor activity during 2h in the open field test of control offspring and AB offspring at P28–P32. AB offspring show less spontaneous locomotor activity in the entire area of the open field than control offspring throughout the test. **^1^P = 0.0041, ***^2^P = 0.0001 (*t*-test), **^3^P = 0.0016 (Welch’s corrected *t*-test); control, n = 22 from 7 dams; AB, n = 18 from 5 dams. (**d**) Locomotor activity in the inside area (area A+B) of the field in the open field test. **^1^P = 0.0045 (Welch’s corrected *t*-test), **^2^P = 0.0011, ****^3^P<0.0001, **^4^P = 0.0012 (Mann–Whitney test). (**e**) Locomotor activity in the innermost area (area A) of the field. *^1^P = 0.0356, ***^2^P = 0.0004, ***^3^P = 0.0009 (Mann–Whitney test). AB offspring prefer to explore the peripheral area of the field compared with control offspring. (**f**) Number of times of rearing, considered to be an exploratory behavior evoked by novel stimuli, observed during 2 h in the open field test. **P = 0.0023 (Welch’s corrected *t*-test).

**Fig 5:**
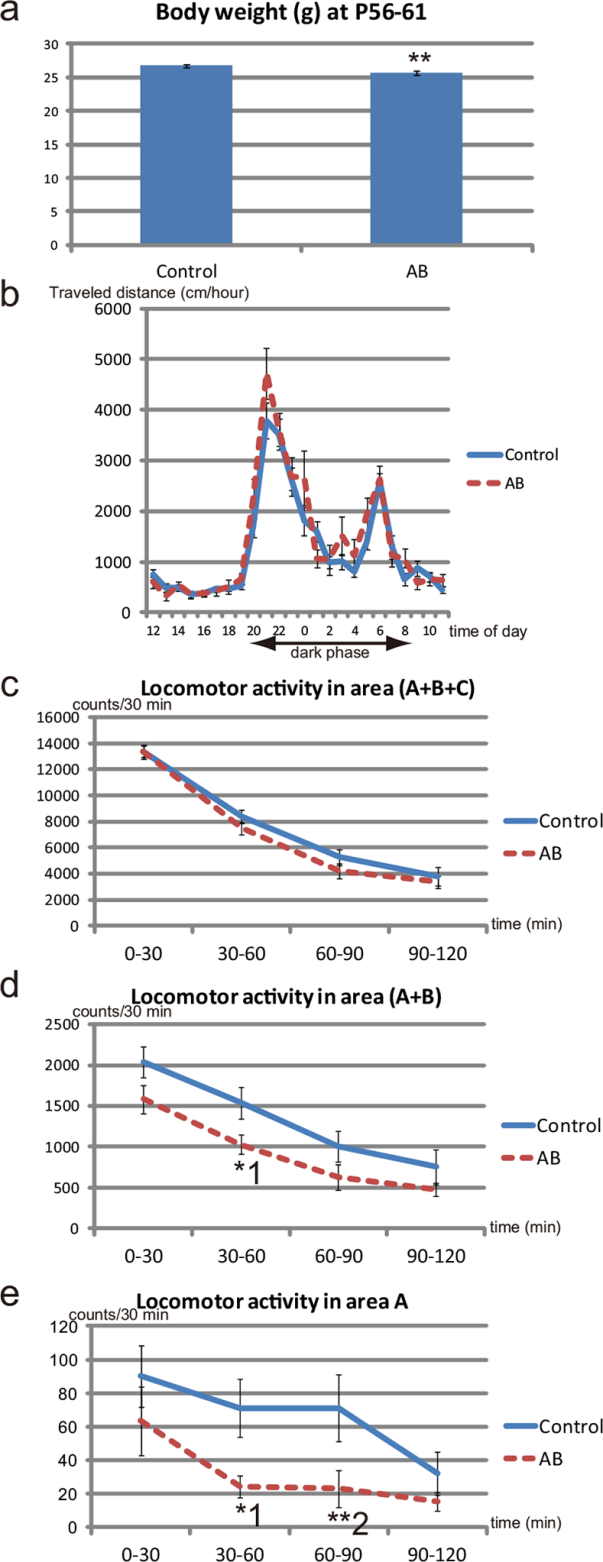
Body weight and behaviors of AB offspring at PW7–PW8. (**a**) Body weight at P56–P61 among offspring from control dams (control offspring; Control) and offspring from AB-treated dams (AB offspring; AB). **P = 0.0054 (unpaired *t*-test); control, n = 28; AB, n = 18. (**b**) The 24-h cycle activity in a familiar environment for control offspring and AB offspring at P56–P61. No significant difference between the two groups is observed at any time-point; control, n = 28 from 8 dams; AB, n = 18 from 5 dams. (**c**) Locomotor activity during 2h in the open field test of control offspring and AB offspring at P54–P59. No difference is observed in the locomotor activity of the two groups; control, n = 21 from 7 dams; AB, n = 18 from 5 dams. (**d**) Locomotor activity in the inside area (area A+B; see Fig 3D) of the field in the open field test at P54–P59. *^1^P = 0.0319 (*t*-test). (**e**) Locomotor activity in the innermost area (area A; see Fig 3D) of the open field at P54–P59. *^1^P = 0.0358, **^2^P = 0.0095 (Mann–Whitney test). AB offspring still prefer to explore the periphery of the field compared with control offspring at P54–59. The mice that had been subjected to behavioral tests at PW4 (Fig 4) were subjected to behavioral tests at PW7–PW8, although one mouse failed to be subjected to 24hr home cage activity test at PW7–PW8, and another mouse failed to be subject to the open field test at PW7–PW8 (please refer to S8 Data).

**Fig 6:**
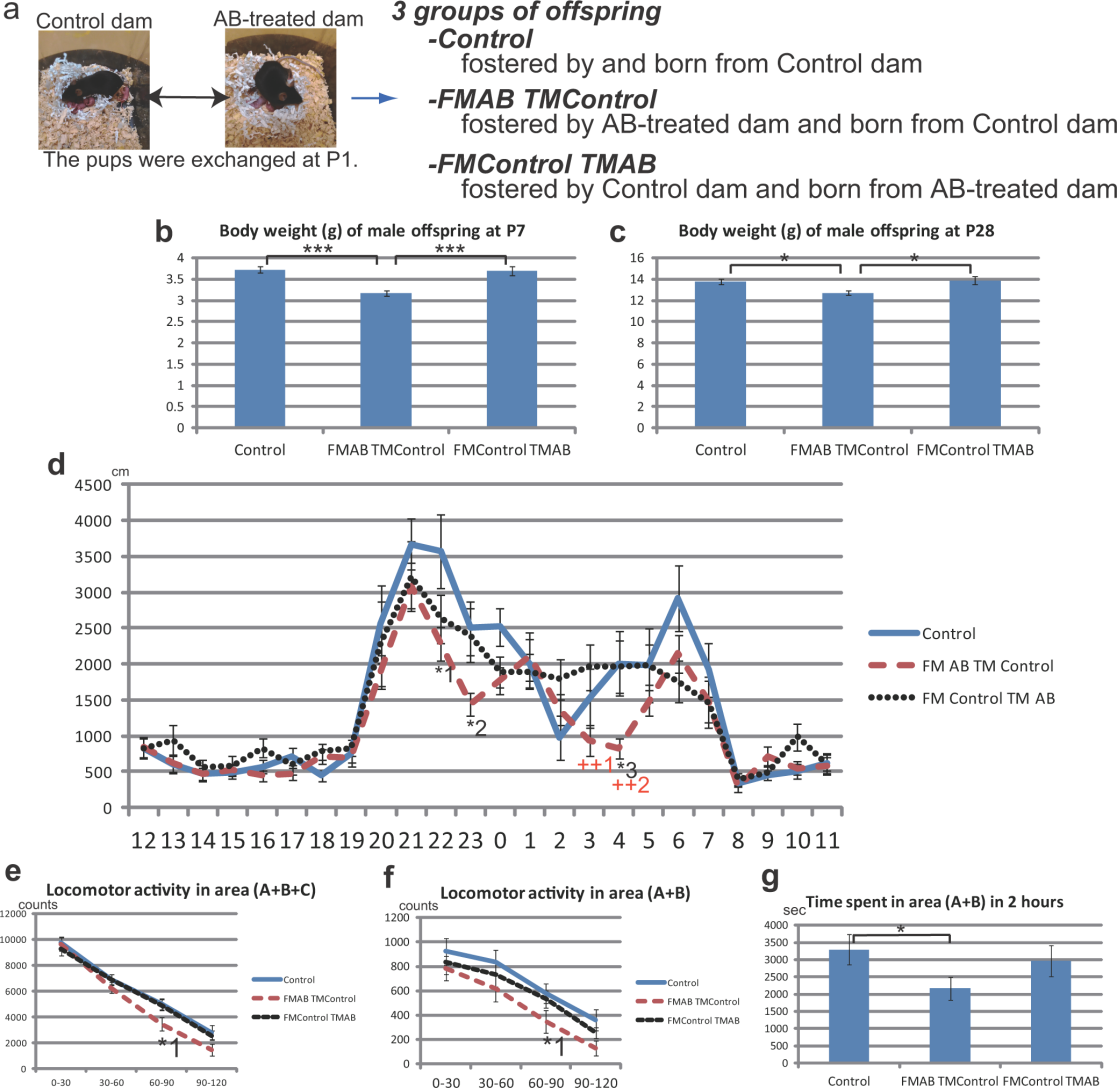
Phenotypes of AB offspring can be rescued by fostering of the offspring by control dams. (**a**) Outline of the experiment in which we exchanged pups at P1 between control and AB-treated dams. We designated offspring fostered by AB-treated dams and born from control dams as ‘FMAB TMControl’ and those fostered by control dams and born from AB-treated dams as ‘FMControl TMAB’. (**b**) Body weight at P7 of control, FMAB TMControl, and TMControl FMAB male and female offspring. ***P<0.001 (Kruskal–Wallis test followed by a post–hoc Dunn’s multiple-comparisons test); control, n = 27 from 7 dams; FMAB TMControl, n = 35 from 9 foster dams; FMControl TMAB, n = 22 from 8 foster dams. (**c**) Body weight at P28 of control, FMAB TMControl, and TMControl FMAB male offspring. *P<0.05 (one-way ANOVA followed by a post–hoc Tukey–Kramer multiple-comparisons test); control, n = 27 from 7 dams; FMAB TMControl, n = 36 from 9 foster dams; FMControl TMAB, n = 21 from 8 foster dams. (**d**) The 24-h cycle activity in a familiar environment of control, FMAB TMControl, and TMControl FMAB offspring at P28–P33. FMAB TMControl offspring exhibit low activity throughout the dark phase (20:00 to 08:00). *^1^P<0.05(22:00–22:59), *^2^P<0.05 (23:00–23:59) *^3^P<0.05 (04:00–04:59), FMAB TMControl vs. control; ^++1^P<0.01 (03:00–03:59), ^++2^P<0.01 (04:00–04:59; Kruskal–Wallis test followed by a post–hoc Dunn’s multiple-comparisons test), FMAB TMControl vs. FMControl TMAB.; control, n = 21 from 6 dams; FMAB TMControl, n = 28 from 7 foster dams; FMControl TMAB, n = 21 from 8 foster dams. (**e**) Locomotor activity during 2h in the open field test at P28–P34. FMAB TMControl offspring exhibit lower spontaneous activity in an unfamiliar environment, particularly in the last 60 min. *^1^P<0.05 (Kruskal–Wallis test followed by a post–hoc Dunn’s multiple-comparisons test), FMAB TMControl vs. control; control, n = 25 from 6 dams; FMAB TMControl, n = 28 from 7 foster dams; FMControl TMAB, n = 21 from 8 foster dams. (**f**) Locomotor activity in the inside area (area A+B; see Fig 3D) of the open field. FMAB TMControl offspring prefer to engage in exploratory behavior in the peripheral area. *^1^P<0.05 (Kruskal–Wallis test followed by a post–hoc Dunn’s multiple-comparisons test), FMAB TMControl vs. control. (**g**) Total time spent in the inside area (area A+B; see Fig 3D) during 2 h in the open field test. FMAB TMControl offspring spend less time in the central area of the open field. *P<0.05 (Kruskal–Wallis test followed by a post–hoc Dunn’s multiple-comparisons test), FMAB TMControl vs. control.

SPF timed-pregnant C57BL/6J mice were purchased from SLC Japan (Hamamatsu, Japan). Embryonic and postnatal stages were calculated with the day of vaginal plug detection as E0 and with the day of birth of offspring as P0. We attempted to deplete mice of their gut microbiota by providing a combination of non-absorbable AB according to the published protocols [4, 5, 13]. Non-absorbable AB become concentrated in the gastrointestinal tract, thereby reducing the production of intestinal microbiota with limited serum concentrations [14, 15]. Pregnant C57BL/6J mice received a solution of non-absorbable AB, comprising 5 mg/ml neomycin trisulfate salt hydrate (Sigma–Aldrich, St. Louis, MO, USA; N6386-25G), 5 mg/ml bacitracin (Sigma–Aldrich; B0125-1250KU), 1.25 μg/ml pimaricin (Sigma–Aldrich; P9703-25MG; 5 mg/ml solution in acetic acid used as a stock), and 0.075% (v/v) acetic acid (Nacalai Tesque, Kyoto, Japan; 08885–45) dissolved in drinking water, which was administered by voluntary drinking on E9–E16 [5]. Intraperitoneal administration of the non-absorbable AB solution to SPF mice or oral administration of the non-absorbable AB solution to germ-free mice did not induce any apparent effects [5], implying that the non-absorbable AB solution used in this study had minimal collateral effects. Control mice received normal drinking water. Throughout the period of AB administration, mice consumed AB solution constantly (S1 Fig). Control and AB-treated mice gave birth to pups and nurtured them *ad libitum*. The litter sizes of control and AB-treated dams, sex ratios of their offspring at P7, and scatter plots showing the relationships between litter sizes and body weights of individual offspring are shown in S2 Fig. Offspring from control and AB-treated mice were weaned from their mothers at P23, and 2–5 sex-matched mice from control or AB-treated mice were housed in a single cage. The offspring from control dams and AB-treated dams were never cohoused in the same cage. A sex bias has been observed in several neurodevelopmental disorders in humans, in which males are at greater risk than females [16, 17]. Therefore, we determined to perform the measurements of body weights and behavioral tests on male offspring only after weaning. In the experiments shown in Figs 1–4 and S2–S4 Figs, we measured the body weight of a male mouse at the time when it was subjected to its initial behavioral test, Therefore, the body weights of some mice were measured at P28, while those of other mice were measured at P29. The total body weight data of the male offspring from control dams and AB-treated dams obtained at P28 or at P29 are shown in S2 Data labelled as ‘Body weight litter size P28–29’. Mice were tested in a single behavioral test per day in postnatal week (PW) 4 or PW 7–8. Most of the mice were subjected to three (experiments shown in Figs 4 and 5 and S3 and S5 Figs) or two (experiments shown in Fig 6 and S7 Fig) behavioral tests, although some mice were subjected only to one or two of the tests. Lists of mice subjected to the behavioral tests are shown in S8 Data. In the ‘cross-fostering’ experiments, pups were swapped between a control mother and an AB-treated mother at P1. We made it a rule to exchange the whole litters from a control mother and the whole litters from an AB-treated mother when swapping pups, with an exceptional case in which a control mother fostered the pups from two AB-treated dams (see raw data in S5 Data). The pups were then nurtured by their foster mother, weaned from their foster mother, and housed as described above. The litter sizes of the group of offspring, sex ratio of the groups of offspring at P7, and scatter plots showing the relationships between litter sizes and body weights of individual offspring in the groups in the cross-fostering experiments are shown in S6 Fig. In the cross-fostering experiments, the body weights of all male mice at PW4 were measured specifically on P28 unless they were not subjected to behavioral tests on P28. The average room temperature fluctuates seasonally in our animal facility (from 23°C in winter to >26°C in summer) because of influences from the outside temperature, which may have affected the offspring growth and basal level of general locomotor activity. Therefore, care was taken that experiments for comparison of specific groups were carried out during the same season. Specifically, the experiments shown in Figs 1–5 and S2–S5 Figs were mainly performed during July–August (tests for a few mice were performed in November–December), while those shown in Fig 6 and S6 and S7 Figs were performed in December–January.

### 24-h home cage activity test

Spontaneous activity in a familiar environment was assayed using a home cage activity monitoring system (O’Hara & Co., Tokyo, Japan), which included a home cage (29 × 18 × 12 cm) and an infrared video camera. A 12-h/12-h light/dark cycle using LED illumination was programmed (08:00–20:00, light period; 20:00–08:00, dark period). Each mouse was separately housed in a cage by 09:30, and images from each cage were captured at a rate of 1 frame/s for 27 h until 12:30 of the next day. Travelled distance per hour was analyzed for 24 h (12:31–12:30 of the next day).

### Open field test

Open field test was performed to measure the locomotion in a novel open field using SCANET MV-40 (MELQUEST Co., Toyama Japan) [18]. The open field consisted of a square box of 48 cm per side with infrared sensors arranged at 6-mm intervals for the automatic detection of activity. Areas A and B were defined as squares of 7.8 cm or 24.6 cm, respectively inside the open field (Fig 4B). Individual mice were placed at the right and front corner of the open field at the initiation of the testing session and allowed to freely explore the field for 2 h. The recordings were performed from 15:00 for 2 h after the mice in the original cages had been placed in the test room for 30 min for acclimatization to the environment.

### Social interaction test

Social behavior was examined using a three-chamber social interaction test system (TimeCSI2; O’ Hara & Co.) to measure how long the subject mice spent with stranger mice. The apparatus was a rectangular, three-chambered box divided by two transparent walls with small doorways allowing access to each chamber (refer to Fig b in S4 Fig). The following procedure with six sessions was recorded as shown in Fig a in S4 Fig: (1) ‘Empty’ session, each subject mouse was placed in the middle chamber at the initiation of the test and allowed to explore all parts of the area freely for 5 min; (2) ‘Stranger’ session, after habituation, an unfamiliar mouse (Stranger 1: C57BL/6N female) was placed in the wire cage in the right chamber, while the wire cage in the left chamber remained empty, and the subject mouse was placed in the middle chamber and allowed to explore for 5 min; (3–5) ‘Familiar’ session 1–3, the ‘Stranger 1’ mouse was kept in the wire cage in the right chamber, and exploration with free access to every chamber by the subject mouse was repeated for 5 min; (6) ‘2nd stranger’ session, the ‘Stranger 1’ mouse in the wire cage in the right chamber was swapped with another unfamiliar mouse, ‘Stranger 2’, and the subject mouse was placed in the middle chamber and allowed to explore for 5 min. Throughout sessions 2–6, the wire cage in the left chamber remained empty. A digital video was recorded for each session, and the amounts of time spent in the area around each cage were automatically analyzed using the TimeCSI_Online program.

### Gut bacterial flora analyses

Analyses of the mouse gut flora using mouse fecal specimens were outsourced to TechnoSuruga Laboratory (Shizuoka, Japan). Bacterial DNA extraction from fecal specimens was performed as previously described [19]. The copy numbers of bacteria contained in the fecal specimens were calculated from a standard curve based on the known copy numbers of *Escherichia coli* JCM 1649^T^ by quantitative real-time PCR of the 16S rRNA gene using the PCR primers: 341f, 5′-CCTACGGGAGGCAGCAG-3′, and 534r, 5′-ATTACCGCGGCTGCTGG-3′ [20]. The bacterial flora composition was examined by terminal restriction fragment length polymorphism (T-RFLP) analyses [21]. The detailed protocols for these analyses were described previously [22, 23]. In brief, the 16S rRNA gene was amplified by PCR from fecal bacterial DNA using fluorescently labelled primers 516F (5′-TGCCAGAGCCGCGGTA-3′) and 1510R (5′-GGTTACCTTGTTACGACTT-3′). The purified PCR products were digested with *Bsl*I, and the T-RF lengths were analyzed with an ABI PRISM 3130*xl* genetic analyzer (Applied Biosystems, Foster City, CA, USA). The fragment sizes were estimated using GeneMapper software (Applied Biosystems). The major T-RFs with similar sizes of 1–3 bp were summarized according to operational taxonomic units (OTU). OTU data were used to identify phylotypes by matching with those predicted from various phylotypes in a database based on the literature ([24]; http://www.tecsrg.co.jp/t-rflp/t_rflp_hito_OTU.html). Relative abundance of bacteria belonging to each phylotype was determined as the percentage of individual OTU per total OTU areas. A two-tailed unpaired *t*-test, two-tailed unpaired Welch’s corrected *t*-test or two-tailed unpaired Mann–Whitney test was used to compare mean values of the relative abundance of each bacterial phylotype between two groups as described below [22, 25, 26]. Cluster analyses were performed using Gene Maths (Applied Maths, Kortijk, Belguim) based on the *BsI*I T-RFLP patterns. Distances were calculated to determine the similarity among samples, and were represented by dendrograms. Pearson’s similarity coefficient analysis and unweighted pair-group methods with arithmetic means (UPGMA), and Dice’s similarity coefficient analysis and the Ward algorithm were used to prepare dendrograms.

### Statistical analyses

Values were expressed as means ± standard error of the mean (SEM). Statistical analyses were conducted using GraphPad InStat version 3.10 (GraphPad Software, Inc., La Jolla, CA, USA). A two-tailed unpaired *t*-test, two-tailed unpaired Welch’s corrected *t*-test, or two-tailed unpaired Mann–Whitney test was used to compare the mean values between two groups following the wizard of the GraphPad InStat program [27]. When comparing three groups, one-way ANOVA followed by a post-hoc Tukey–Kramer multiple-comparisons test or a Kruskal–Wallis test followed by a post-hoc Dunn’s multiple-comparisons test was chosen for the analyses following the wizard of the GraphPad Instat program. Values of P < 0.05 were considered to indicate statistical significance.

## Results

### Effects of administration of non-absorbable AB to pregnant mice on E9–E16

During development, neurogenesis from neural stem cells starts to occur around at E10 and lasts until E17–E18 in the rodent telencephalon [28, 29]. We administered non-absorbable AB to pregnant mice on E9–E16 to observe the effect of perturbation of the maternal gut microbiota during pregnancy on neural development of offspring. During this period of AB administration, weight gain in AB-treated mice was reduced compared with that in control pregnant mice (Fig 1A). By day 4 of the 7-day period of AB administration, the mean copy number of the microbiota in feces of AB-treated mothers was reduced to 2.317% of that in control mothers (Fig 1B). Examination of the bacterial flora composition by T-RFLP analysis showed that the gut flora composition was affected by AB treatment (Fig 1C). The relative abundance of *Clostridium* cluster XI was significantly lower in AB treated mice than in control mice (Fig 1C). The relative abundances of the bacterial order *Lactobacillales* and the bacterial family *Clostridium* subcluster XVIII were lower in AB-treated dams, although the differences did not reach the statistical siginificane (Fig 1C). The relative abundance of the bacterial family *Prevotella* was decreased to zero in AB-treated dams, although the statistical evaluation could not be performed on this difference because standard deviation of the data from AB-treated dam was totally zero (Fig 1C). Cluster analysis based on the results of T-RFLP analyses by two different methods revealed that control dams and AB-treated dams were mostly grouped in different clusters (Fig 1D and 1E).

### Effects on the body weight and the gut microbiota of offspring by administration of non-absorbable AB to pregnant dams

The sex ratios of offspring born from control dams (control offspring) and those born from AB-treated dams (AB offspring) did not differ (Fig a in S2 Fig). The mean litter size was slightly lower in AB-treated mice (5.50 ± 0.49) than that in control mice (6.63 ± 0.29), although the difference did not reach statistical significance (P>0.05; S2B Fig). The mean body weight of AB offspring was lower than that of control offspring at P7 (Fig 2A; the mean body weights of female offspring at P7 are shown in Fig c in S2 Fig). Although AB offspring had reduced the gap in body weight by PW4, the difference in body weight between control and AB offspring remained significant at P28 (Fig 2B). No difference was observed for the estimated copy numbers of fecal bacteria between AB offspring and control offspring at P24, which is 1 day after weaning from dams (Fig 3A). The bacterial flora composition analyses showed that the relative abundance of the bacterial order *Lactobacillales* was lower in AB offspring (20.7±3.4%) than in control offspring (42.1±6.2%), while the relative abundance of the bacterial family *Clostridium* subcluster XIVa was higher in AB offspring (34.2±5.0%) than in control offspring (16.4±3.3%; Fig 3B). However, the mean relative abundances of the other bacterial families in control offspring were similar to those in AB offspring (Fig 3B). A cluster analysis of fecal microbiota compositions performed by two different methods revealed contrasting branching patterns of dendrograms (Fig 3C and 3D), supporting the idea that the gut flora compositions in control offspring and AB offspring differed in some parts and resembled each other to a certain extent in other parts. Overall, we conclude that perturbation of the maternal microbiota during pregnancy leads to moderate changes in the gut flora composition at P24, primarily driven by alterations in the relative abundances of the bacterial order *Lactobacillales* and the bacterial family *Clostridium* subcluster XIVa.

### Effects on the behaviors of offspring at PW4 after administration of non-absorbable AB to the dams during pregnancy

To determine whether administration of non-absorbable AB to pregnant dams affected the brain development of their offspring, we performed behavioral tests on offspring from control and AB-treated dams at PW4. The 24-h home cage activity tests revealed that the activity level in a familiar environment in the dark phase was generally lower in AB offspring than that in control offspring (Fig 4A). The reduced activity in AB offspring compared with that in control offspring was also observed in the open field test to measure spontaneous locomotor activity in a novel environment (Fig 4C; Fig a in S3 Fig). Notably, AB offspring engaged less and spent a shorter amount of time undertaking exploratory locomotive behavior in the inside area, compared with control offspring (Fig 4D and 4E; Fig b in S3 Fig). Rearing is considered to be an exploratory behavior induced by novel stimuli [30]. Consistent with the reduced exploratory locomotive behavior in AB offspring, the frequency of rearing behaviors in a novel environment was lower in AB offspring than in control offspring (Fig 4F). Next, we performed three-chambered social interaction tests on control and AB offspring. The control offspring and AB offspring spent a similar amount of time around the cage housing the stranger mice (S4 Fig), suggesting that the two types of offspring showed similar levels of interest in the stranger mice in the three-chamber social interaction tests. These results suggest that AB administration to dams during pregnancy to perturb the maternal gut microbiota leads to alterations in the behaviors of their offspring at PW4.

### Effects on the behaviors of offspring at PW7–PW8 after administration of non-absorbable AB to their dams during pregnancy

We examined whether the low body weight and behavioral characteristics of AB offspring at PW4 could be observed in adults at PW7–PW8. The difference in mean body weight between control offspring and AB offspring was smaller at PW8 than at PW4, although the difference remained significant (Fig 5A). AB offspring exhibited a similar level of spontaneous locomotor activity in both a familiar environment and an unfamiliar environment compared with control offspring (Fig 5B and 5C; Figs a, b in S5 Fig), although AB offspring still tended to explore the center of the unfamiliar environment for less time than control offspring at PW7– PW8 (Fig 5D and 5E).

### Fostering of AB offspring by control dams from P1 rescues the phenotypes of AB offspring at PW4

Next, we examined the effects of exchanging pups between control and AB dams at P1 (Fig 6A). Specifically, we compared the phenotypes of offspring fostered by AB dams that were originally born from control dams (FMAB TMControl) and those of offspring fostered by control dams that were originally born from AB-treated dams (FMControl TMAB) with those of control offspring. Notably, the mean body weight of FMControl TMAB offspring remained slightly lower than that of control offspring, whereas the body weight of FMAB TMControl offspring was even lower than those of control offspring and FMControl TMAB offspring at P7 (Fig 6B; the body weights of female offspring at P7 are shown in S6C Fig). This low body weight of the FMAB TMControl group compared with the other groups was still observed at P28, and even at P76–P77 (Fig 6C; Fig a in S7 Fig). The 24-h cycle rhythm of spontaneous locomotor activity in a familiar environment of FMControl TMAB offspring was mostly similar to that of control offspring (Fig 6D). FMAB TMControl offspring exhibited a lower level of spontaneous locomotor activity in the dark phase than did control offspring (Fig 6D). The level of locomotor activity, spatial preference for locomotion, and frequency of rearing behavior in an unfamiliar environment of FMControl TMAB offspring were similar to those of control offspring (Fig 6E–6G; Figs b, c in S7 Fig). The locomotor activity of FMAB TMControl offspring in the open field test had a tendency to be lower than those of control offspring and FMControl TMAB offspring (Fig 6E; Fig b in S7 Fig). The frequency of rearing behavior in FMAB TMControl offspring was significantly less than those in the other groups (S7C Fig). FMAB TMControl offspring tended to engage in less exploratory behavior in the inside area of the open field compared with control offspring and FMControl TMAB offspring (Fig 6F and 6G). These findings show that low body weight, low level of spontaneous behavior in both familiar and unfamiliar environments, and spatial preference for locomotion in the inside area, which were observed in AB offspring at PW4, were recovered to a considerable extent by fostering of the offspring by control dams from P1. Furthermore, control offspring fostered by AB-treated dams exhibited similar phenotypes to those of AB offspring at PW4.

## Discussion

Accumulating evidence has demonstrated the importance of the gut microbiota in shaping brain development and behavior [31–33]. The aim of this study was to explore the contribution of the maternal gut microbiota to the brain development of offspring by examining whether perturbation of the maternal gut microbiota during pregnancy can affect the development of the central nervous system of offspring by performing behavioural analyses on the offspring born from AB treated mice. Our data showed that administration of non-absorbable AB to dams during pregnancy to perturb the maternal gut microbiota led to reduced body weight and alterations in the behavior of their offspring at PW4. It is evident that the gut microbiota has a balanced compositional signature that provides the host with health benefits [6]. Several factors and events can potentially affect the gut microbial composition, including host genetics, dietary shifts, infection, disease, surgery, drug treatments (including AB-treatment), and transplantation [1, 2, 6, 34]. Although our study did not fully elucidate the detailed mechanisms underlying the development of the phenotypes of AB offspring, our results imply that it is important to take care of the maternal gut microbiota during perinatal periods for healthy development of offspring.

In our study, T-RFLP analyses of the gut flora composition showed that the relative abundance of the bacterial order *Lactobacillales* was lower in AB offspring than in control offspring, while the relative abundance of the bacterial family *Clostridium* subcluster XIVa was higher in AB offspring than in control offspring at P24. The question then arises as to whether these alterations in the gut flora profile of AB offspring are associated with the abnormal behavior of AB offspring. One of the experiments to answer this question involves examination of whether oral treatment of AB offspring with a species of the bacterial order *Lactobacillales* can rescue the low body weight and abnormal behavior of AB offspring. It was recently reported that administration of *Lactobacillus fermentum* NS9 reduced anxiety-like behavior in rats that were also administered with ampicillin [35], implying that oral treatment of AB offspring with a species of the bacterial order *Lactobacillales* is promising. Other important questions are how the alterations in the gut flora composition at P24 are generated and how long these alterations continue. The relative abundance of the bacterial order *Lactobacillales* was also lower in the feces of AB-treated dams than in those of control dams, although no statistical significance was obtained in the difference. It is possible that the alteration in the gut microbiota profiles of AB-treated dams is associated with that in AB offspring. Performance of comparative studies on the temporal profiles of the colonization of the gut bacterial microbiota of control offspring and AB offspring after birth and the temporal profiles of the compositions of the gut microbiota in control and AB-treated dams after the end of AB administration would give answers to this question.

In this study, the phenotypes of offspring born from AB-treated dams could be rescued to a considerable extent at PW4 by fostering of the offspring by control dams from P1. Offspring from control dams fostered by AB-treated dams (FMAB TMControl) exhibited similar phenotypes to those of offspring born from AB-treated dams (Fig 6). These findings suggest that the effects of administration of non-absorbable AB to pregnant mice during E9–E16 to perturb the maternal gut microbiota may influence the development of offspring, principally after birth. Notably, the mean body weight of offspring from control dams fostered by AB-treated dams became even lower than that of control offspring at P7, implying that there is a critical effect of administration of non-absorbable AB to perturb the maternal gut microbiota during pregnancy on the postnatal development of offspring, particularly in the first week after birth. The gut microbiota contributes significantly to host nutrient metabolism, such as dietary fibre metabolism, lipid uptake and deposition, breakdown of indigestible polysaccharides to absorbable monosaccharides, and vitamin synthesis [1, 36, 37]. Epidemiological data in clinical studies in animals have revealed that maternal perinatal undernutrition increases the subsequent risk of deficits in the brain and behavioral function in offspring [38–44]. Food restriction of rat dams during perinatal periods induced hyperactivity of the hypothalamic pituitary adrenal (HPA) axis with increased plasma glucocorticoid level, and decreased the level of locomotor activity in the offspring [43, 44]. Notably, maternal food restriction only during lactation period influenced the activity of the HPA axis, suggesting that this early postnatal period is a sensitive target of maternal undernutrition [42]. Furthermore, cross-fostering experiments suggested that nursing the offspring born from food-restricted dams by control mothers during lactation normalized the alteration in decreased locomotor activity of offspring [44]. Maternal perinatal undernutrition could influence the composition of breast milk. For example, it has been reported that maternal undernutrition during perinatal periods leads to reduced concentrations of leptin (a peptide hormone to regulate metabolism and appetite) in breast milk [44]. One possible mechanism for the postnatal effect of AB administration during pregnancy is that the AB-treated dams were in poor nutritional condition throughout the perinatal periods because of the perturbed condition of the gut microbiota, leading to the production of low-nutrition breast milk with an untoward influence on the postnatal development of their offspring [45–47]. Another possible mechanism is related to the source of the microbiota in offspring as mentioned above. The infant gut is considered to be mostly sterile at birth [2, 48]. Colonization of the gut with microbes begins immediately after birth. Infants born by vaginal delivery have microbial communities that resemble those found in the maternal vaginal microbiota [2]. Microbes from the mouth and skin of mothers can also be transferred vertically to offspring [49]. Furthermore, breast milk contains particular oligosaccharides that act as prebiotics for *Bifidobacteria*, and is also known to contain multiple bacteria species that are thought to be transferred to offspring [47, 49, 50]. Thus, mothers are the first and most important source of microbes for their offspring [1, 2, 48]. As the maternal gut microbiota was disturbed by AB treatment during the perinatal periods, the colonization of gut microbes in offspring after birth would be disrupted, which could possibly lead to disruption of the brain development of offspring after birth because the vertical transmission of maternal microbes to offspring and the subsequent bacterial colonization of the neonatal gut overlap with a critical period of brain development [51–53]. Another possible mechanism is associated with the maternal stress caused by administration of non-absorbable AB to pregnant dams to perturb the maternal gut microbiota and the subsequent nurturing behavior in the postnatal period. Germ-free mice exhibit exaggerated hypothalamic-pituitary-adrenal (HPA) response to stress, suggesting that the gut microbiota has a role in the development of the HPA axis [3, 6]. Administration of non-absorbable AB to pregnant dams to perturb the gut microbiota may induce increased responses to stress in the dams, which could possibly lead to significant alterations in maternal care after the birth of their offspring [54]. Clinical researches in human have shown that maltreatment early in childhood can cause a lifelong reduction in cognitive performance, alteration in stress response, and an increase in the risk of mood- and stress-related disorders [55, 56]. Animal models of aberrant maternal care or maternal separation have also demonstrated alterations in behavior and brain structure [57–59]. Therefore it may be possible that perturbation of the maternal gut microbiota during pregnancy affect maternal care after birth of offspring, resulting in alteration of behavior of offspring.

In the current study, we used T-RFLP method for the analyses of the composition of gut microbiota. This method is widely used in investigating the gut microbiota of the rodents [22, 60–67]. However, this method has limitations that analyses are based on the database of human gut microbiota with rather low resolution. We have to take into consideration these limitations when we evaluate the results of the comparisons of the gut microbiota profiles between control dams and AB-treated dams or between control and AB offspring. In the future experiments, we should have to undertake sequencing-based methods in combination with appropriate metagenomic analyses to identify the phylogenic profiles of the gut microbiota of AB dams and AB offspring with higher resolution [68, 69].

In this study, we did not control the number of animals to be fostered by a mother throughout the experiments. Control and AB-treated mice gave birth to pups and nurtured them *ad libitum*. In cross-fostering experiments, whole litters of a control mother and whole pups of an AB-treated mother were exchanged with an exception. Litter sizes of dams vary to a considerable extent both in control dams and in AB-treated dams (Fig b in S2 Fig). Normalizing the numbers of pups fostered by a mother to a certain number in each litter was practically difficult under normal experimental conditions in which we had to produce as many pups as possible with limited numbers of pregnant dams. No statistical significance was obtained in the mean litter size between in control dams (6.63 ± 0.29) and in AB-treated dams (5.50 ± 0.50; Fig a in S2 Fig). The difference in the numbers of offspring fostered by each mother in cross-fostering experiment was not statistically significant among control (6.29 ± 0.28), FMAB TMControl (7.22 ± 0.40) and FMControl TMAB offspring (5.00 ± 0.82), either (Fig b in S6 Fig). Distributions of body weights in each litter size imply that there is no apparent effect of litter size on the body weight at P7 or P28 (Figs d-i in S2 Fig and Figs d-l in S6 Fig). However, it might be expected that we evaluate more correctly the effect of perturbation of the maternal gut microbiota or the effect of cross-fostering on the development of offspring by minimizing the differences in the numbers of offspring fostered by a mother. We might have to consider this in the future experiment.

In the open field test, the level of anxiety in a novel environment can be measured by the preference to stay near a wall [70]. Notably, AB offspring engaged less and spent a shorter amount of time undertaking exploratory locomotive behavior in the inside area, compared with control offspring. Widely used mouse assays for anxiety-related behaviors include the elevated plus–maze, elevated zero–maze, light–dark exploration, marble burying, shock–probe burying, stress–induced hyperthermia and the operant Vogel thirsty–lick conflict test [70, 71]. In future studies, we would ascertain if AB offspring exhibit apparent anxiety-related behaviors using some of these behavioral tests.

The results of our study suggest that administration of non-absorbable antibiotics to pregnant mice to perturb the maternal gut microbiota is associated with alterations in offspring behavior, although the detailed underlying mechanisms have not been elucidated. It can be expected that such hidden aspects of the functions of the gut microbiota in the maintenance of healthy maternal metabolism and/or healthy mother–infant relationships during the perinatal periods will be revealed by investigation of the detailed mechanisms whereby the behavioral phenotypes of AB offspring are developed.

## Supporting Information

S1 Data Raw data related to Fig 1. (XLSX)

S2 Data Raw data related to Figs 2 and 3 and S2 Fig. (XLSX)

S3 Data Raw data related to Fig 4 and S3 Fig. (XLSX)

S4 Data Raw data related to Fig 5 and S5 Fig. (XLSX)

S5 Data Raw data related to Fig 5 and S6 and S7 Figs. (XLSX)

S6 Data Raw data related to S1 Fig. (XLSX)

S7 Data Raw data related to S4 Fig. (XLSX)

S8 Data Lists of mice subjected to the behavioral tests. (XLSX)

S1 Fig Volume of solution consumed by pregnant dams administered non-absorbable AB. The volume of drinking water containing non-absorbable AB consumed by AB-treated dams is slightly less than that of normal drinking water consumed by control dams during the initial periods (days 1–2) of the 7-day period of AB administration. However, the solution intake of AB-treated dams become more similar at days 3–6 of administration, and is increased at day 7 of administration, compared with that of control dams. ****^1^P<0.0001, *^2^P = 0.0298 (*t*-test); control, n = 15; AB, n = 14.
(EPS)

S2 Fig Sex ratios and litter sizes of offspring born from control dams and AB-treated dams, and body weights of female offspring at P7. (**a**) Relative abundance of male and female offspring born from control dams and AB-treated dams, examined at P7. (**b**) Distribution of litter sizes of offspring born from control dams and AB-treated dams. Bars indicate the mean litter sizes of control offspring (6.63±0.29) and AB offspring (5.50±0.50), respectively. No statistical significance is observed for the difference in litter sizes between control offspring and AB offspring (P = 0.0548, Mann–Whitney test). Control, n = 24; AB, n = 12. (**c**) Body weights at P7 of female offspring from control dams (control offspring; Control) and AB-treated dams (AB offspring; AB). No statistical significance is obtained in the difference between control offspring and AB offspring (P = 0.1403, *t*-test). Control, n = 17 from 7 dams; AB, n = 10 from 4 dams. (**d**) Distribution of body weights at P7 of male offspring born from control dams in each litter size. n = 27 in 7 litters. (**e**) Distribution of body weights at P7 of male offspring born from AB-treated dams in each litter size. n = 15 in 4 litters. (**f**) Distribution of body weights at P7 of female offspring born from control dams in each litter size; n = 17 in 7 litters. (**g**) Distribution of body weights at P7 of female offspring born from AB-treated dams in each litter size. n = 10 in 4 litters. (**h**) Distribution of body weights at P28 of male offspring born from control dams in each litter size. n = 15 in 4 litters. (**i**) Distribution of body weights at P28 of male offspring born from control dams in each litter size. n = 9 in 3 litters.
(EPS)

S3 Fig Phenotypes observed in the open field test for control offspring and AB offspring at P28–P32. (**a**) Total locomotor activity during 2h in the open field test. The locomotor activity of AB offspring is less than that of control offspring in the open field test. **P = 0.001 (*t*-test). (**b**) Total time spent in the inside area (area A+B; see Fig 3D) during 2 h in the open field test. AB offspring spend less time in the inside area. ***P = 0.0004 (Mann–Whitney test); control, n = 22 from 7 dams; AB, n = 18 from 5 dams.
(EPS)

S4 Fig No differences in social interaction between control offspring and AB offspring at P28–P32. (**a**) Schematic figure showing the six sessions of the social interaction test using the three-chambered apparatus. (1) ‘Empty’ session, each subject mouse was placed in the middle chamber at the initiation of the test and allowed to explore all parts of the area freely for 5 min; (2) ‘Stranger’ session, after habituation, an unfamiliar mouse (Stranger 1: C57BL/6N female) was placed in the wire cage in the right chamber, while the wire cage in the left chamber remained empty, and the subject mouse was placed in the middle chamber and allowed to explore for 5 min; (3–5) ‘Familiar’ session 1–3, the ‘Stranger 1’ mouse was kept in the wire cage in the right chamber, and exploration with free access to every chamber by the subject mouse was repeated for 5 min; (6) ‘2nd stranger’ session, the ‘Stranger 1’ mouse in the wire cage in the right chamber was swapped with another unfamiliar mouse, ‘Stranger 2’, and the subject mouse was placed in the middle chamber and allowed to explore for 5 min. Throughout sessions 2–6, the wire cage in the left chamber remained empty. **(b)** Snapshot of the ‘Stranger’ session in the three-chamber social interaction test. A subject mouse stays around a stranger mouse in the right cage. (**c**) Graph showing the time spent around the right cage area in the ‘Empty’, ‘Stranger’, ‘Familiar 1–3’, and ‘2nd stranger’ sessions. Control offspring and AB offspring spend a similar amount of time in the right cage area in each session; control, n = 27 from 7 dams; AB, n = 15 from 4 dams. (**d**, **e**) Representative 5-min traces for the movements of mice placed in the area of the three-chamber social interaction test for control offspring (**d**) and AB offspring (**e**) in the ‘Stranger’ session.
(EPS)

S5 Fig Phenotypes observed in the open field test of control offspring and AB offspring at P54–P59. (**a**) Total locomotive activities during 2 h in the open field. The locomotor activities during 2 h in the open field test are similar for control offspring and AB offspring at P54–P59. (**b**) Numbers of rearing events during 2 h in the open field test observed in control offspring and AB offspring; control, n = 21 from 7 dams; AB, n = 18 from 5 dams.
(EPS)

S6 Fig Sex ratios and litter sizes of control, FMAB TMControl, and FMControl TMAB offspring, and body weights of female offspring at P7. (**a**) Relative abundances of male and female in control, FMAB TMControl and FMControl TMAB offspring, examined at P7. (**b**) Distribution of litter sizes (number of offspring simultaneously fostered by a mother) of control, FMAB TMControl and FMControl TMAB offspring. Bars indicate the mean litter sizes of control offspring (6.29 ± 0.28), FMAB TMControl offspring (7.22 ± 0.40) and FMControl TMAB offspring (5.00 ± 0.82) respectively. No statistical significance was observed for the difference in litter sizes among control, FMAB TMControl and FMControl TMAB offspring (P>0.05, Kruskal–Wallis test). Control, n = 7; FMAB TMControl, n = 9; FMControl TMAB, n = 8. (**c**) Body weight at P7 of control, FMAB TMControl, and TMControl FMAB female offspring. ***P<0.001 (Kruskal–Wallis test followed by post–hoc Dunn’s multiple-comparisons test); control, n = 17 from 7 dams; FMAB TMControl, n = 30 fostered by 8 foster dams; FMControl TMAB, n = 17 fostered by 7 foster dams. (**d**) Distribution of body weights at P7 of male control offspring in each litter size; n = 27 fostered by 7 dams. (**e**) Distribution of body weights at P7 of male FMAB TMControl offspring in each litter size. n = 36 fostered by 9 foster dams. (**f**) Distribution of body weights at P7 of male FMControl TMAB offspring in each litter size; n = 22 fostered by 8 foster dams. (**g**) Distribution of body weights at P7 of female control offspring in each litter size; n = 17 fostered by 7 foster dams. (**h**) Distribution of body weights at P7 of female FMAB TMControl offspring in each litter size; n = 29 fostered by 8 foster dams. (**i**) Distribution of body weights at P7 of female FMControl TMAB offspring in each litter size; n = 18 fostered by 7 foster dams. (**j**) Distribution of body weights at P28 of male control offspring in each litter size; n = 27 fostered by 7 foster dams. (**k**) Distribution of body weights at P28 of male FMAB TMControl offspring in each litter size; n = 36 fostered by 9 foster dams. (**l**) Distribution of body weights at P28 of male FMControl TMAB offspring in each litter size; n = 22 fostered by 8 foster dams.
(EPS)

S7 Fig Phenotypes of offspring in cross-fostering experiments. (**a**) Mean body weight of control, FMAB TMControl and FMControl TMAB offspring at P76–P77; **P<0.01 (Kruskal-Wallis test followed by a post hoc Dunn's Multiple Comparisons test); control, n = 20 from 5 dams; FMAB TMControl, n = 28 from 7 foster dams; TMAB FMControl, n = 15 from 6 foster dams. (**b**) Total locomotor activity during 2 h in the open field test at P28–P34. FMAB TMControl offspring exhibit reduced locomotor activity in the open field test compared with control and FMControl TMAB offspring, although the differences do not achieve statistical significance (P>0.05, FMAB TMControl vs. Control; P>0.05, FMAB TMControl vs. FMControl TMAB, Kruskal–Wallis test followed by a post–hoc Dunn’s multiple-comparisons test). (**c**) FMAB TMControl offspring show less rearing behaviors during 2h in the open field test at P28–P34. **P<0.01 (Kruskal–Wallis test followed by a post–hoc Dunn’s multiple-comparisons test); control, n = 25 from 6 dams; FMAB TMControl, n = 28 from 7 foster dams; FMControl TMAB, n = 21 from 8 foster dams.
(EPS)
